# Does Smartphone Use Make Older Adults Feel Younger? A Cross-Sectional Online Survey during the COVID-19 Pandemic

**DOI:** 10.3390/ijerph20031710

**Published:** 2023-01-17

**Authors:** Tomoko Ikeuchi, Sakiko Itoh, Hiroyasu Miwa, Kentaro Watanabe, Tomoko Wakui

**Affiliations:** 1 Human Care Research Team, Tokyo Metropolitan Institute of Gerontology, Tokyo 173-0015, Japan; 2 Department of Genome Informatics, Graduate School of Medicine, Osaka University, Osaka 565-0871, Japan; 3 Service Value Augmentation Research Team, Human Augmentation Research Center, National Institute of Advanced Industrial Science and Technology, Chiba 277-0882, Japan

**Keywords:** subjective age, ICT use, psychological well-being, older adults

## Abstract

Subjective age (i.e., how old one feels) has been found to be a biopsychosocial marker of aging. This study examined the associations between subjective age and the frequency of information and communication technology (ICT) usage by older adults. Data were collected via an online survey conducted in 2020 during the COVID-19 pandemic. The study analyzed responses from participants aged 65 to 89 (*M* = 71.9, *SD* = 3.9) who resided in Japan (*N* = 1631, 52.8% female). Subjective age was indexed by asking participants to specify in years how old they felt. Proportional discrepancy scores (PDS) were calculated to indicate younger or older subjective age and were used as an independent variable. Participants were asked about the frequency of computer, smartphone, flip phone, tablet, and social networking service (SNS) use. Two-thirds of the participants (63.6%) reported feeling younger than their actual age. Nearly 90% reported using computers for more than 2–3 days a week, while 64.3% reported smartphone use, 22.9% reported flip phone use, and 36.6% reported SNS use. Logistic regression analyses revealed that a lower PDS (i.e., feeling younger) was associated with a significantly higher frequency of smartphone use (OR: 0.77; 95% CI: 0.60, 0.98) after adjusting for potential confounders. No such association was found for computer, flip phone, tablet, or SNS use. Our study found that feeling younger was associated with a higher frequency of smartphone use. The daily use of smartphones may have helped older adults stay in touch with family and friends and obtain the information that they needed, which may have contributed to better psychological well-being outcomes, especially during the COVID-19 pandemic.

## 1. Introduction

The coronavirus disease 2019 (COVID-19) pandemic has forced many older adults to self-isolate by remaining at home and avoiding social contact with family and friends [1]. As a result, time alone, with the physical absence of others, increased significantly for many older adults. However, the average older person was already spending half of his or her waking hours alone before the COVID-19 pandemic. A study of American time use reported that older adults spend an increasing amount of time alone as they age: those aged between 65 and 85+ years spend 7.4 to 8.4 hours per day by themselves, and 10 to 20% of them spend all day alone. Furthermore, older adults who live alone spend almost 11 hours alone in their activities, which is about twice as much time as those who live with a partner [2].

Living alone is associated with spending time alone for older adults, and a great amount of time alone could reflect social isolation. As the number of one-person households headed by an older person is increasing across countries that have a rapidly growing proportion of the older population, social isolation among older adults compounds the cost of eldercare [3]. For example, in Japan, nearly 29% of those aged 65 years and older lived alone in 2019 [4]. In the United States, more than 40% of those aged 89 years and older lived alone in 2018 [5]. Living alone has been found to be associated with negative outcomes, such as cognitive decline and lower levels of well-being [6]. Likewise, greater average amounts of alone time were found to be associated with higher levels of loneliness and depression and lower levels of well-being [2,7].

As a measurement of well-being, subjective age, i.e., how old or young a person feels, has been the focus of much attention [8]. Ayalon et al. [9] found that changes in subjective age correspond to changes in subjective experiences among older adults. They reported that a decrease in loneliness was associated with an accelerated decrease in subjective age, whereas an increase in depressive symptoms was associated with an accelerated increase in subjective age. Feeling younger than their chronological age (i.e., younger subjective age) has been reported to be associated with better health and well-being [8]. Subjective age can be a reflection of individuals’ physical health [10], mental health [11], age identity, and age stereotypes or beliefs [8].

Age stereotypes directed toward oneself in old age can be viewed as self-perceptions of aging [12]. The stereotype embodiment theory proposes that stereotypes internalized across the lifespan lead to self-definitions and affect health outcomes. Better health and cognitive functioning and greater longevity have been associated with positive self-perceptions of aging [13], whereas negative self-perceptions of aging are associated with poor physical and mental health [12]. Kotter-Grühn et al. [8] elucidated that self-perceptions of aging and subjective age are the constructs encompassed by the umbrella term ‘subjective aging.’ Feeling younger or older than one’s chronological age can be closely related to self-perceptions of aging. For example, feeling older can reflect negative self-perceptions of aging.

For older adults who live alone and spend a considerable amount of time alone, information and communication technologies (ICTs) can play a role in helping them stay connected with family and friends and the resources they need [14]. Having a sense of being connected with others can lead to better well-being for those who spend most of their time alone. However, older adults are less likely than younger adults to use technology in general [15] or to share the perception that ICTs can improve the quality of daily life. Those who do not use ICT often do not see the need for it [16]. Some older adults can be passive, especially in regard to new and unfamiliar technologies. Those who are not familiar with ICTs must first adopt ICTs to become proficient in their use. Older adults with negative self-perceptions of aging may be hesitant to adopt unfamiliar ICTs. Some may think they are “too old” to learn new things. If older adults with negative attitudes toward their own aging fail to master the use of ICTs, they may perceive themselves as an old person with a diminished sense of mastery [17].

### Research Purpose and Hypothesis

In the present study, we examined the associations between subjective age and frequency of ICT usage of older adults during the COVID-19 pandemic. We hypothesized that feeling younger would be associated with higher frequencies of ICT use. We predicted that more frequent use of ICTs would be related to better well-being, i.e., younger subjective age, as a result of being connected to friends and family and having access to the resources they need while self-isolating due to the pandemic. To test our prediction, we asked the participants who use ICTs to indicate their reasons for using ICTs as well as the frequency of their use of ICTs.

## 2. Materials and Methods

### 2.1. Data and Participants

This cross-sectional study used data collected via an online survey conducted in August 2020 during the COVID-19 pandemic. At that time, the number of COVID-19 cases in Japan was rapidly increasing, and people were asked by the Japanese government to refrain from unnecessarily leaving their homes. Participants were members of online survey panels recruited by a survey company in Japan. To reduce the bias effects of age, sex, and region, eligibility was calculated based on the population structure of Japan using the 2020 Basic Resident Registration System [18]. Eligible participants were invited to participate in the survey and given incentives for participation. The survey company limited the number of invitations to the panel by restricting participation to the month of August 2020. All participants resided in Japan at the time of participation and had access to the internet to allow participation in the online survey.

### 2.2. Measurement

ICT use. Participants were asked about the frequency of their use of personal computers (PCs), smartphones, flip phones, tablets, and social networking services (SNS) such as Twitter, Facebook, Instagram, LINE, etc. They rated the frequency of their use on a 5-point Likert scale (1 = daily, 2 = 2–3 days a week, 3 = a few times a month, 4 = a few times a year, 5 = none). Those who answered that they use it daily or 2–3 days a week were categorized into the “frequent use” group, while those who used it less frequently than a few times a month were categorized into the “infrequent use” group. The binary value of frequent (= 1) and infrequent (= 0) use was used as a dependent variable. Participants who indicated that they use these ICTs were further instructed to select all that applied from the following list of reasons for using them: life necessity, trustworthy, inexpensive, useful, and/or amusing.

Subjective age. Subjective age was indexed by asking participants to specify in years how old they felt at the moment. Participants who answered that they were younger than 18 years old were excluded (*n* = 7). A proportional discrepancy score (PDS) was calculated by an equation ((subjective age - chronological age)/chronological age) to obtain a measure of subjective age in proportion to chronological age [19,20]. Negative scores indicate that participants feel younger than their chronological age (e.g., a value of −0.20 indicates that a participant feels 20% younger). For our logistic analyses, we divided the participants into two groups using the PDS: (1) those whose scores show a negative value (i.e., feeling younger than their chronological age) and (2) those whose scores show a value of zero (i.e., feeling the same age as their chronological age) or a positive value (i.e., feeling older than their chronological age). The binary value that was created based on the PDS was used as an independent variable.

Control variables. A set of control variables was included in the analysis models: sex (1 = male, 2 = female); living arrangement (0 = not living alone, 1 = living alone); subjective health (1 = not good, 2 = good/very good), which was assessed with a single item of an individual’s own health condition; and level of education (1 = less than high school, 2 = high school or junior college, 3 = college graduate or above).

### 2.3. Sample Characteristics

**Figure 1:**
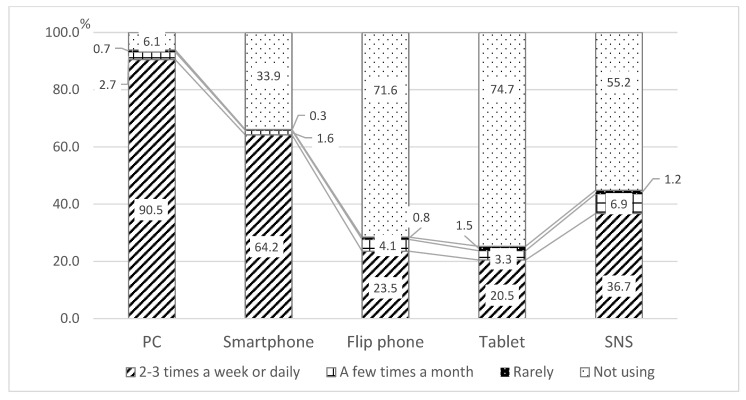
Frequencies of ICT use.

The individuals who completed the survey provided information about their subjective age, their use and frequency of use of ICT, including PCs, smartphones, flip phones, tablets, and SNS, and potential covariates, including sex, living arrangement (i.e., living alone), subjective health, and education levels. The study analyzed the data of 1631 participants aged 65 to 89 years. The mean age was 71.9 (*SD* = 3.9) years, and 54.3% were female. Nearly 78% of the participants reported feeling younger than their actual age, and 22.1% reported that they felt the same as or older than their actual age. As shown in Figure 1, more than 90% of participants reported using PCs several times a week or every day, 64.2% reported using smartphones, 23.5% reported using flip phones, 20.5% reported using tables, and 36.7% reported using SNS, i.e., Twitter, Facebook, Instagram, or LINE. More than 67% reported that their health was good or very good, while 32.9% reported that their health was not good. Nearly 40% identified as a college graduate or above, 57.6% graduated from a high school or junior college, and 3% reported less than a high school education. The characteristics of the participants are presented in Table 1.

### 2.4. Statistical Analysis

We examined the independent associations of subjective age, using the PDS, with the frequency of ICT use after adjusting for potential confounders using logistic regression analysis. The analysis was performed separately for each dependent variable, i.e., PC, smartphone, flip phone, tablet, and SNS. Potential confounders were evaluated for collinearity, including sex, living arrangement, subjective health, and education levels. Statistical analyses were conducted using Stata 16 [21]. A *p*-value of less than 0.05 was considered indicative of statistical significance.

## 3. Results

The subjective age variable was expressed in continuous numbers from 18 to 98 years of age (*M* = 65.7, *SD* = 7.7). Chronological age was significantly correlated with subjective age (*r* = 0.467, *p* ≤ 0.001). Comparing subjective age with chronological age, 77.9%, 17.4%, and 4.7% of the participants reported that they felt younger than their chronological age, felt the same age, and felt older, respectively. The scores of PDS ranged from −0.76 to 0.36, and the mean score was −0.09 (*SD* = 0.09); i.e., participants felt 9% younger on average. The PDS was significantly correlated with subjective age (*r* = 0.886, *p* ≤ 0.001).

**Figure 2:**
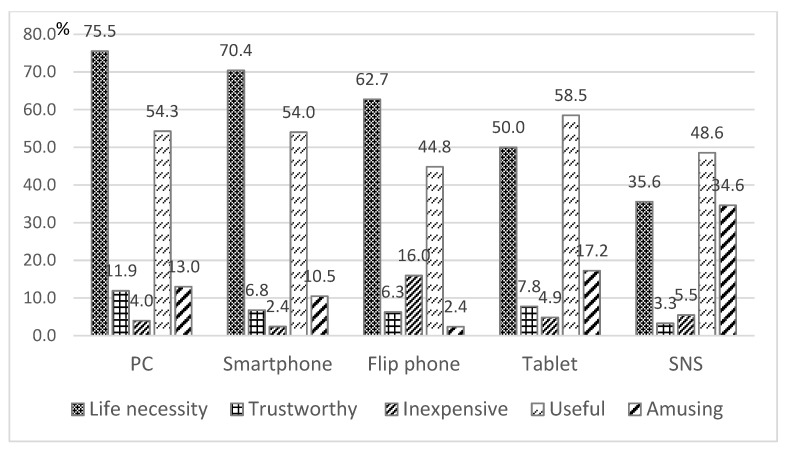
Reasons for ICT use.

Figure 2 shows the percentage of responses for the different reasons for ICT use among those who indicated that they use ICTs. For the use of PCs, smartphones, and flip phones, ‘life necessity’ was the most common reason, followed by ‘useful.’ For the use of tablets and SNS, ‘useful’ was the most common reason, followed by ‘life necessity.’ For the use of SNS, ‘amusing’ was the third most common reason. ‘Trustworthy’ and ‘inexpensive’ were less-common reasons for the use of these ICTs. We also measured the correlations among the frequencies of the type of ICTs used and found that PC use was negatively correlated with smartphone (*r* = −0.098, *p* ≤ 0.001) and tablet use (*r* = −0.162, *p* ≤ 0.001). Smartphone use was negatively correlated with flip phone use (*r* = −0.629, *p* ≤ 0.001) and positively correlated with tablet (*r* = 0.100, *p* ≤ 0.001) and SNS use (*r* = 0.346, *p* ≤ 0.001).

Table 2 presents the results of the logistic regression analysis. Controlling for sex, living alone, subjective health, and education level, the results showed that subjective age equal to or older than chronological age was associated with a significantly lower odds ratio (OR) of smartphone use (0.77, 95% confidence interval = [0.60, 0.98]). No associations were found between subjective age and the use of PCs, flip phones, tablets, or SNS. Meanwhile, there were significant associations between the female sex and a lower frequency of use for PC (0.36, [0.24, 0.54]) and a higher frequency of use for SNS (1.45, [1.17, 1.81]). Moreover, higher levels of education were associated with significantly higher ORs for PC use (3.30, [1.44, 7.54] for a college graduate or above) and SNS use (2.37, [1.13, 4.96] for high school or junior college; 3.75, [1.77, 7.92] for a college graduate or above). Furthermore, living alone was associated with a significantly lower OR of smartphone use (0.74, [0.57, 0.97]). Subjective health showed no associations with the frequency of ICT use.

## 4. Discussion

The present study examined the associations of subjective age with the frequency of ICT use among older adults using an online survey during the COVID-19 pandemic. We found that younger subjective age was associated with a higher frequency of smartphone use. More than 70% of respondents who are smartphone users indicated that it is a necessity in their daily lives. Given that smartphones are communication technologies that allow users to communicate anytime and anywhere, daily necessities may contribute to the quality of their relationships and a sense of belonging [22]. That would have been particularly important during the pandemic when people were encouraged to stay home and maintain physical distance from others as much as possible. Prior to the pandemic, many older adults attempted to prevent adverse health outcomes through regular social participation and daily physical activity [23]. However, when those daily routines became extraordinary, the value of ICT use became significant.

Moreover, a recent survey of Japanese older adults’ use of ICT services found that with the widespread use of smartphones among older adults, there has been a particularly marked increase in information retrieval and social media transmission [24]. Our study also found that smartphone use and SNS use were positively correlated. It is reasonable to think that older adults who use smartphones are increasingly searching for information and possibly sharing it with family and friends through social media. According to socioemotional selectivity theory (SST) [25], when time horizons (i.e., future time) are perceived as long, priority is given to goals such as increasing knowledge and acquiring information to prepare for the future to come. Older adults who frequently use smartphones to search for information could be looking further into the future than they are looking into the present and, therefore, may feel younger in subjective age.

Furthermore, the generational digital divide still exists, and it is reported that older adults use technologies far less often than younger people [17]. Older adults who are proficient in using a smartphone in their daily lives may have perceived themselves as being not much different from young and middle-aged individuals in terms of their technological ability. Stereotypical beliefs affecting ICT use in older adults are common, which may have contributed to the younger subjective age associated with the higher frequent use of smartphones found in our study.

Although there are various barriers that prevent older adults from using ICTs, increasing numbers of older adults across countries have been exposed to and use ICTs in their daily lives [14]. However, there is still a great need to enhance the accessibility of ICT literacy for older adults. A recent study reported that the most common reason for smartphone ownership among older Japanese adults was that it was “recommended by a family member” [24]. Family members and friends who are ICT literate can volunteer to help their older family members and friends. Meanwhile, our present study found that living alone was associated with a lower frequency of smartphone use. Older adults are often comfortable with the use of ICT once someone helps them with the set-up process. The initial step of performing the set-up process and having someone to call who is willing to help will likely encourage older adults who are not ICT literate [26] and who may even have a certain amount of ICT-related phobia. However, older adults who live alone may be less likely than those who live with others to receive such assistance when they need it. Therefore, the availability of family or friends who are ICT literate may influence the use of ICT by older adults.

The present study has several limitations. First, due to the cross-sectional correlational design of this study, we were unable to eliminate the possibility of reverse causation. It is necessary to examine the causal relationship using a longitudinal study design in future research to determine whether the use of ICT works to lower the subjective age of older adults or whether younger subjective age is conducive to the use of ICT. Second, because our data were collected during the COVID-19 pandemic, which has impacted the physical and psychological health of millions of people worldwide [27], the subjective age of participants may have been negatively affected by the circumstances as well.

Our data were collected using an online survey. Therefore, it is a reasonable possibility that the results of this study may be skewed toward those who use ICTs more frequently than average. However, a Japanese national survey conducted in 2021 using a random location quota sampling method reported that approximately 86% of Japanese adults aged 60+ and 67% of those aged 70+ were using smartphones. Of these, 82.6% of those aged 60+ and 60% of those aged 70+ used the social media service called Line, which is one of the most popular messaging applications in Japan [28]. In the present study, 64% reported using a smartphone several times a week or daily. This figure is compatible with the national survey that was conducted one year later than the present study.

## 5. Conclusions

The majority of older adults in Japan experience good health beyond the age of 70 years, with healthy life expectancy in 2019 at 72.7 years for men and 75.4 years for women [29]. Along with the increase in healthy life expectancy, the use of ICTs, such as smartphones and mobile messaging apps, by older adults has increased rapidly. Our study found that feeling younger was associated with a higher frequency of smartphone use. For older adults who are relatively healthy and active, smartphones can enrich their lives and contribute to better well-being by connecting them with family and friends. General ICTs have proven effective in alleviating the social isolation and loneliness of older adults [14]. The challenge is, however, how to increase ICT literacy among the older adult population. There are a considerable number of older adults who do not use ICTs in their daily lives. How can they learn new technologies without feeling that they are “too old” to do so? With the increasing costs of eldercare in many industrialized countries due to social isolation and the associated deterioration of mental health, strategies to alleviate social isolation need to be further explored. The results of this study demonstrate the potential of ICT to mitigate these social challenges.

## Figures and Tables

**Table 1:** Characteristics of participants (*N* = 1631).

Variable			
Age, Mean (*SD*)		71.9	(3.9)
Sex	Male, *n* (%)	770	(47.2)
	Female, *n* (%)	861	(52.8)
Frequency of ICT use			
PC	Low, *n* (%)	155	(9.5)
	High, *n* (%)	1476	(90.5)
Smartphone	Low, *n* (%)	584	(35.8)
	High, *n* (%)	1047	(64.2)
Flip phone	Low, *n* (%)	1247	(76.5)
	High, *n* (%)	384	(23.5)
Tablet	Low, *n* (%)	1297	(79.5)
	High, *n* (%)	334	(20.5)
SNS	Low, *n* (%)	1032	(63.3)
	High, *n* (%)	599	(36.7)
Subjective age			
Younger than actual age, *n* (%)		1270	(77.9)
Same as or older than actual age, *n* (%)		361	(22.1)
Proportional discrepancy scores (PDS), *Mean (SD)*		−0.09	(0.09)
Living alone, *n* (%)		272	(16.7)
Subjective health			
Not good, *n* (%)		536	(32.9)
Good/very good, *n* (%)		1095	(67.1)
Highest level of education			
Less than high school, *n* (%)		49	(3.0)
High school or junior college, *n* (%)		939	(57.6)
College graduate or above, *n* (%)		643	(39.4)

Note. For frequency of ICT use, ‘Low’ indicates a frequency of less than a few times a month/year or none, and ‘High’ indicates a frequency of several times a week or more/every day.

**Table 2:** Independent associations of PDS with frequency of ICT use among 1631 older adults.

	PC	Smartphone	Flip Phone	Tablet	SNS
	Adjusted+ OR (95% CI)	Adjusted+ OR (95% CI)	Adjusted+ OR (95% CI)	Adjusted+ OR (95% CI)	Adjusted+ OR (95% CI)
*n*	1631	1631	1631	1631	1631
Subjective age					
Younger than actual age	1 (ref)	1 (ref)	1 (ref)	1 (ref)	1 (ref)
Same as or older than actual age	1.13 (0.73–1.73)	0.77 (0.60–0.98) *	1.13 (0.85–1.49)	0.97 (0.72–1.31)	0.79 (0.61–1.02)
Sex					
Male	1 (ref)	1 (ref)	1 (ref)	1 (ref)	1 (ref)
Female	0.36 (0.24–0.54) **	0.99 (0.79–1.23)	1.02 (0.80–1.31)	1.20 (0.92–1.55)	1.45 (1.17–1.81) **
Living alone					
No	1 (ref)	1 (ref)	1 (ref)	1 (ref)	1 (ref)
Yes	1.08 (0.70–1.67)	0.74 (0.57–0.97) *	1.07 (0.79–1.45)	0.91 (0.66–1.27)	1.21 (0.92–1.58)
Subjective health					
Not good, *n* (%)	1 (ref)	1 (ref)	1 (ref)	1 (ref)	1 (ref)
Good/very good, *n* (%)	1.21 (0.83–1.75)	1.00 (0.80–1.23)	1.08 (0.84–1.40)	1.05 (0.80–1.37)	1.05 (0.84–1.31)
Highest level of education					
Less than high school, *n* (%)	1 (ref)	1 (ref)	1 (ref)	1 (ref)	1 (ref)
High school or junior college, *n* (%)	1.55 (0.73–3.32)	0.95 (0.52–1.75)	1.22 (0.60–2.49)	0.81 (0.41–1.63)	2.37 (1.13–4.96) *
College graduate or above, *n* (%)	3.30 (1.44–7.54) **	0.90 (0.48–1.67)	1.16 (0.56–2.40)	1.04 (0.51–2.10)	3.75 (1.77–7.92) **

Note. ** *p* < 0.01, * *p* < 0.05. Higher ORs indicate a higher frequency of ICT use. Logistic regression models were run separately. +Adjusted for sex, living alone, subjective health, and highest level of education.

## Data Availability

The data supporting the findings of this study are available from the corresponding author upon reasonable request.
